# Association of internet gaming disorder with impulsivity: role of risk preferences

**DOI:** 10.1186/s12888-023-05265-y

**Published:** 2023-10-16

**Authors:** Lin Zhu, Yuqiong Zhu, Shuxuan Li, Yulian Jiang, Xian Mei, Yuting Wang, Dengxian Yang, Jing Zhao, Linlin Mu, Wenjuan Wang

**Affiliations:** 1 School of Mental Health, Bengbu Medical College, Bengbu, Anhui 233030 China; 2 School of Qian Xuesen College, Xi’an Jiaotong University, Xi’an, Shanxi 710049 China

**Keywords:** Internet gaming disorder, Impulsivity, Risk preferences, College students

## Abstract

**Background:**

Internet gaming disorder (IGD) is a formal mental disorder leading to personal and social impairment. Although it shares similar physical and psychosocial effects to substance use disorder, the psychological mechanisms underlying IGD remain unclear, although several researches have made significant contributions to its understanding. This study aims to elucidate the correlation between IGD, impulsive personality and risk preference of medical college students in China, from a questionnaire-based investigation.

**Methods:**

Based on the cluster random sampling method, a questionnaire survey was conducted among medical college students in Northern Anhui, China from September 3 to October 27, 2020. The questionnaires included the Internet Gaming Disorder Scale (IGD-20), Chinese revised of Barratt Impulsiveness Scale Version 11 (BIS-11), and risk appetite index (RPI). Perform independent sample t-tests, analysis of variance (ANOVA), correlation analysis, and moderating effect analysis using SPSS 23.0. *P* < 0. 05 is considered statistically significant.

**Results:**

624 participants completed the survey, including 257 males (41.19%) and 367 females (58.81%). All participants were between 18 and 24 years. We found that in IGD and its six different dimensions and RPI, males scored significantly higher than females. Additionally, our finding revealed there is statistical significance in IGD and impulsiveness between gaming group with game time greater than or equal to 4 h and non-gaming group. The IGD and its six different dimensions, among which all except for mood modification are positively correlated with impulsiveness and RPI. Mediating effects indicate that RPI plays a partial mediating role between motor impulsiveness and IGD.

**Conclusion:**

The findings shows that there is a certain relationship between impulsivity and RPI, as well as IGD and its dimensions. RPI may be a mediator between impulsivity and IGD, and men have higher IGD. The findings supported the compensatory hypothesis. These findings may contribute to further research and development of intervention and prevention measures for IGD.

**Supplementary Information:**

The online version contains supplementary material available at 10.1186/s12888-023-05265-y.

## Background

The rapid evolution of the Internet over the past decades has led to a surge in the number of people accessing it. According to the 48th China Internet development statistical report: as of June 2021, the number of online game users in China reached 509 million, accounting for 50.4% of the overall Internet users. Generally speaking, moderate gaming can meet the psychological needs of some teenagers for belonging, curiosity, and self actualization; Some sports and puzzle games have a positive effect on the healthy growth of teenagers. However, excessive game addiction leads to internet gaming disorder [1]. Gaming disorder is an addiction involving excessive focus on online or offline internet gaming, leading to a disregard for regular activities and interests, loss of control, and severe impairment in social functioning. The Diagnostic and Statistical Manual of Mental Disorders (DSM-5) first included gaming disorder in its appendix in 2013 [2]. In 2018, the 11th edition of the International Classification of Diseases (ICD-11) officially defined gaming disorder as a mental disorder resulting from addictive behaviors [3].

A recent study revealed that the prevalence of Internet Gaming Disorder (IGD) among the total sample ranged from 0.7–27.5% [4], while the prevalence of IGD among adolescent game players in China was 17.0% [5]. While IGD may only affect a small portion of the population, it is essential to recognize that gaming already significantly impacts various aspects of society. Research has shown that IGD can cause a series of problems such as decreased academic performance, increased stress, depression and anxiety [6–8]. The current literature on gaming addiction suggests that playing an online game may increase the chances of potential addiction compared to an offline game [6, 9]. In terms of neurotransmitters, people with IGD have a lower DAT concentration [10]. In terms of pathophysiology, IGD is related to changes in brain gray matter volume [11]. Using resting state fMRI scans, it is shown that the decreased functional connectivity of IGD is involved in executive function [12]. There are also literature indicating that at different stages, gender may be a potential risk factor for the development of IGD [13]. The occurrence of IGD may be related to family function [14] and early life trauma [15], a longitudinal study shows that good family function predicts lower probability of having internet addiction [16].

Like individuals with substance dependence, impulsivity is considered the most significant trait of gamers with Internet Gaming Disorder [17]. Impulsivity is a major personality trait characterized by the inclination towards quick, impulsive, and unrestrained decisions and actions, regardless of negative consequences. In recent years, impulsivity has been viewed as multidimensional [18, 19], with different subcomponents that have diverse properties and depend on dissociative forms of different cortico-striatal substrates [20]. One form of impulsivity depends on the temporal discounting of reward, while the other depends on motor or response disinhibition. Inhibitive volitional control involves neural circuits of cortical and subcortical mechanisms, especially in the basal ganglia. Impulses caused by chemical dysmodulation may also be included at the level of the striatum. For adolescents with IGD, they tend to prioritize immediate outcomes without considering their risks or negative consequences. Therefore, impulsivity may be a behavioral indicator of IGD [21].

Decision-making plays a significant role in human behavior. In this regard, recent basic research has focused on the three fundamental components of decision-making: judgment, which concerns how individuals forecast the consequences of potential options; preference, which pertains to how individuals evaluate those outcomes; and choice, which encompasses how individuals integrate their judgments and preferences to arrive at a decision. Decision-making can be influenced by individual differences, such as decision-making style, risk propensity, impulsivity, and personality, which may vary depending on the circumstances. Hsee and Weber [22] studied risk preference, pointing out that risk preference refers to a decision-making tendency shown by decision-makers in the face of a situation that includes both risk and security options, which is an important indicator of individual decision-making behavior patterns in non-deterministic risk situations. Risk preferences can be assessed by analyzing task-based decisions made in uncertain situations, as in the Iowa gambling task, or based on estimated risk, as in the adaptive decision-making task [23]. A study on college students revealed that those with Internet addiction scored higher on the Iowa gambling task [24]. IGD patients tend to make intuitive decisions rather than deliberative decisions. This may explain why they continue to participate in internet games despite their negative impact [25]. Risk preference is a relatively stable personality trait that affects the decision-making process and may play a key role in the pathogenesis of IGD [25, 26]. Previous studies have examined the relationship between internet gaming disorder and impulsive personality traits, as well as risk preferences (impulsive choices) separately. However, no existing research has yet identified the specific relationship patterns among these three factors. We hypothesized that risk preference is involved in the mediating effect between impulsivity and IGD.

Herein, we examined the underlying factors and risk elements of online gaming disorder by conducting a questionnaire survey.We sought to investigate and analyze the current state and features of IGD among college students and explore the association between online gaming disorder and impulsivity, as well as risk preference which will help to reveal the neuropsychological mechanism behind online game disorder.

## Methods

### Study design and participants

For this study, an offline questionnaire survey method was utilized. Cluster random sampling was used and 636 questionnaires were distributed, questionnaires with uncompleted answers or suspected unreal answers were excluded. 624 questionnaires with detailed content were collected with the 98.1% effective rate. All participants obtained informed consent. The participants are aged from 18 to 24 years old and reported having experience with using the Internet, including 257 males (41.19%) and 367 females (58.81%). Among them, 269 of whom lived in urban areas (43.11%), 355 in rural areas (56.89%), 213 only children (34.13%), and 411 non only children (65.87%), At the same time, we investigated their parents’ marital status, including normal families, divorced families, reorganized families and single parent families, with normal families accounting for 90.69%, divorced families accounting for 5.3%, and restructured and single parent families accounting for 4.1%. Juniors constituted the most significant proportion of participants (39.90%), followed by freshmen (32.37%) and sophomores (27.72%). In terms of gaming behavior, 8.01% of participants reported spending more than 4 h a day on online games, while 69.07% reported spending less than 4 h a day on online games. Table 1 provides a description of the characteristics of participants.

**Table 1:** Demographic and internet behaviors information of participants (n = 624)

	Variables	Number	Percentage (%)
Grade	Freshman	202	32.37
	Sophomore	173	27.72
	Junior	249	39.9
Gender	Male	257	41.19
	Female	367	58.81
Residence	City	269	43.11
	Town	355	56.89
One-child family and multi-child family	One child	213	34.13
	Multi-child	411	65.87
Parents’ marital status	Normal	566	90.7
	Divorced	33	5.3
	Other	25	4
Game time per day	> 4 h	50	8.01
	< 4 h	431	69.07
	Non	143	22.92
Total time spent playing the game	> 12 months	275	44.07
	< 12 months	349	55.93
Total		624	100

### Measures

#### 20-item internet gaming disorder test (IGD-20)

The IGD-20, developed by American scholars Pontes et al. in 2014 based on the diagnostic criteria of DSM-5 [27], is an important tool specifically designed to measure online gaming disorders. Authorized by Pontus, the original creator of the scale, Qin Lixia and other Chinese scholars translated and revised the Chinese version [28], forming a Chinese questionnaire, which has proved to be effective in distinguishing Chinese college students’ IGD users from other Internet users. The IGD-20 Chinese scale has undergone reliability and validity tests demonstrating its stability and usefulness for measuring IGD in Chinese youth. The IGD-20 scale uses a constitutive model of addiction as its framework, including salience, mood modification, tolerance, withdrawal symptoms, conflict, and relapse. Participants rate their responses on a 5-point Likert frequency scale. If the total score for the IGD-20 items is above 71, then a diagnosis of Internet game disorder may be indicated. Through testing, the scale has good reliability and effectiveness for young Chinese people. The Cronbach’s alpha of the Chinese version of IGD-20 is 0.89.

#### Chinese revised version of Barratt Impulsiveness Scale 11th (BIS-11)

Validation of measurement tools is essential to ensure their effectiveness and reliability across different cultures and clinical samples. The Barratt Impulsiveness Scale [29] consists of three subscales, namely the non-planning impulsiveness, motor impulsiveness, and cognitive impulsiveness subscales; Each subscale has 10 items, with a score range of 1–4 points for each item. The response scale consists of 5 points, ranging from 1 = not at all, 2 = a little bit, 3 = sometimes, 4 = often, to 5 = always. After revision by Li Xianyun et al. [30], the total number of items, subscales, and items in each subscale of the Barratt Impulsiveness Scale in the Chinese version are consistent with the English version, with a score range of 1–5 points for each item. But the items in the unplanned and cognitive impulsivity subscales are all opposite, meaning the corresponding score range is 5 − 1 points. The score of the subscale ranges from 10 to 50, and the higher the respondents’ score, the more impulsive they are. To calculate the scores, the range of scores was converted to percentiles, with the subscale score being calculated as [(sum of item scores − 10) / 40] × 100 and the scale’s total score as the sum of the three subscales divided by 3. Shen Zhihua et al. [31] evaluated the Chinese version of Barratt Impulse Scale with 2295 college students as subjects, the internal consistency between the total scale was 0.85, indicating good reliability and effectiveness.

#### Risk appetite index

This study investigated the calculation method and risk appetite index (RPI) of the risk preference questionnaire developed by Hsee et al. [22]. The RPI value ranges from 1 to 8, with higher values indicating a greater willingness to take risks. For the benefit condition, if the subject chooses a positive benefit in all 7 scenarios, a score of 1 is obtained, while if the subject only chooses gambling in scenario 1 and chooses a positive benefit in the remaining scenarios, a score of 2 is obtained, the others by analogy. A score of 8 is assigned if the subject selects gamble in all scenarios. If the respondent’s answers are illogical, the questionnaire is deemed invalid. Under the loss condition, if the subject chooses a confident return in all scenarios, the score is 1; If the subject only chooses gambling in scenario 7 and has a certain benefit in other scenarios, the score is 2, the others by analogy. Adding the RPI values obtained under both conditions indicated the level of risk preference. This questionnaire and its evaluation methods have been widely used by scholars to measure RPI. The total scale demonstrated a high level of internal consistency, with a coefficient of 0.83.

### Statistical analysis

The data were presented as mean and standard deviation. Perform a test for the normality of the data and confirm that the data follows a normal distribution. Then conduct a parameter test on all the data. Independent sample t-test used for comparison between two data groups. For multiple groups, one-way ANOVA was used, followed by post-hoc comparisons. Correlation analysis was performed to investigate the associations among IGD, impulsivity, and RPI, with a significance level set at *α* = 0.05. Using SPSS 23.0 and plugin PROCESS 4.0 for Moderating effect analysis, and all results were integrated [32]. *P* < 0. 05 was considered statistically significant.

## Results

### Comparison of IGD, BIS, and RPI in gender

**Fig. 1:**
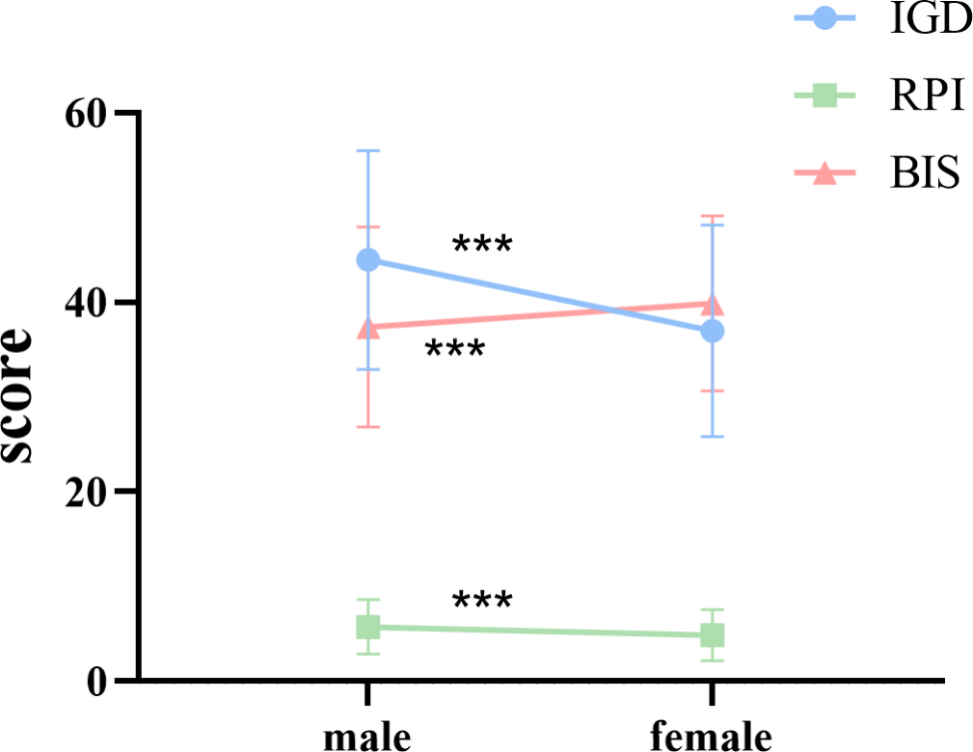
Comparison of IGD, BIS, and RPI in Gender. Comparison of IGD and impulsiveness and RPI between males and females. The y-axis shows mean scores of RPI or IGD or impulsiveness. IGD, internet gaming disorder; BIS, Barratt impulsiveness scale; RPI, Risk appetite index. *P < 0.05, **P < 0.01

The gender differences in IGD and its six different dimensions, impulsivity, and RPI were analyzed using independent samples t-tests (Table 2; Fig. 1). This suggests that in IGD and its six different dimensions and RPI, males scored significantly higher than females. The largest scores and standard deviations were found in the IGD scores, indicating that men were more likely to suffer from internet gaming disorder with more significant individual variability. Women scored significantly higher than men on the Barratt Impulsiveness scale, with higher scores in all three dimensions (motor impulsiveness, cognitive impulsiveness, and non-planning impulsiveness) compared to men. Among them, non-planning impulsiveness factor have the highest scores, and the standard deviation of motor impulsiveness factor is the highest. This indicates that women often exhibit higher levels of unplanned behavior and greater variability in motor impulsiveness.

**Table 2:** Comparison of IGD, BIS, and RPI in gender (M ± SD)

	Gender		*t*	*P*
	Male (*n* = 257)	Female (*n* = 367)		
IGD	44.47 ± 11.55	36.98 ± 11.16	8.13	< 0.001
Salience	6.54 ± 2.44	5.00 ± 1.96	8.43	< 0.001
Mood modification	9.07 ± 2.49	8.12 ± 2.81	4.47	< 0.001
Tolerance	6.81 ± 2.42	5.61 ± 2.28	6.34	< 0.001
Withdrawal symptoms	5.70 ± 2.13	4.63 ± 1.92	6.55	< 0.001
Conflict	10.27 ± 2.98	8.86 ± 2.89	5.90	< 0.001
Relapse	6.07 ± 2.39	4.75 ± 2.06	7.32	< 0.001
Motor Impulsiveness	32.39 ± 15.17	34.10 ± 14.47	-1.42	0.155
Non-planning Impulsiveness	41.02 ± 12.91	44.06 ± 11.82	-3.04	0.002
Cognitive Impulsiveness	38.72 ± 11.62	41.49 ± 9.77	-3.23	0.001
BIS	37.38 ± 10.54	39.88 ± 9.25	-3.14	0.002
RPI	5.72 ± 2.86	4.86 ± 2.68	3.84	< 0.001

Note. IGD, internet gaming disorder; BIS, Barratt impulsiveness scale; RPI, risk appetite index. Values are tested by means of independent samples t-test.

**Table 3:** Comparison of IGD and BIS and RPI in terms of playtime (*M* ± SD)

	Network usage type			*F*	*P*	Post hoc
	Non-game^1^ (*n* = 143)	< 4h^2^ (*n* = 431)	≥ 4h^3^ (*n* = 50)			
IGD	37.45 ± 12.76	40.43 ± 11.21	44.40 ± 13.64	7.09	0.001	2 > 1, 3 > 1
Salience	5.15 ± 2.18	5.69 ± 2.23	6.52 ± 2.82	7.14	0.001	3 > 2 > 1
Mood modification	7.92 ± 3.03	8.65 ± 2.57	9.00 ± 2.86	4.78	0.009	3 > 1, 2 > 1
Tolerance	5.69 ± 2.54	6.20 ± 2.34	6.46 ± 2.53	2.99	0.051	
Withdrawal symptoms	4.79 ± 2.06	5.10 ± 2.04	5.66 ± 2.73	3.40	0.034	3 > 1
Conflict	8.91 ± 3.20	9.47 ± 2.85	10.70 ± 3.41	6.77	0.001	3 > 1, 3 > 2
Relapse	4.99 ± 2.18	5.31 ± 2.28	6.06 ± 2.58	4.13	0.017	3 > 1
BIS	38.94 ± 9.75	38.32 ± 9.62	43.18 ± 11.40	5.52	0.004	3 > 1, 3 > 2
Motor Impulsiveness	32.85 ± 15.23	33.23 ± 14.57	36.40 ± 15.15	2.42	0.090	
Non-planning Impulsiveness	41.773 ± 12.43	42.73 ± 12.21	46.50 ± 13.05	2.76	0.064	
Cognitive Impulsiveness	40.14 ± 10.20	40.03 ± 10.60	43.70 ± 11.92	2.71	0.067	
RPI	4.87 ± 2.76	5.25 ± 2.72	5.84 ± 3.31	2.42	0.090	

Note. IGD, internet gaming disorder; BIS, Barratt impulsiveness scale; RPI, risk appetite index. Values are tested by one-way ANOVA among the groups.

### Comparison of IGD and impulsiveness and RPI in terms of game time

**Fig. 2:**
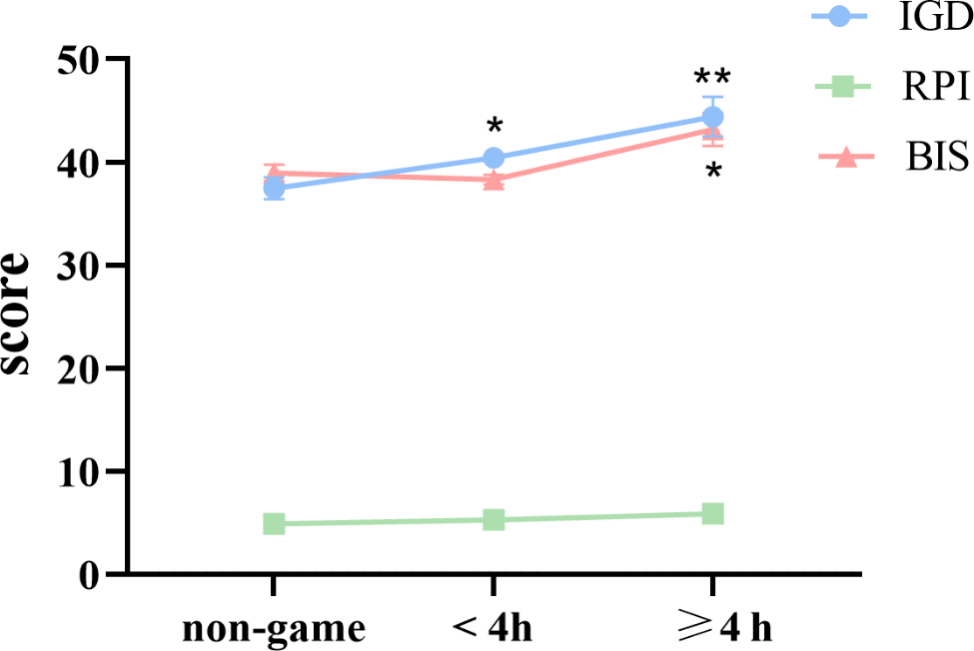
Comparison of IGD, BIS, and RPI in terms of game time. Comparison of IGD, BIS, and RPI between gaming group with a daily time of 4 h or more, gaming group with less than 4 h, and non-gaming group. The y-axis shows mean scores of RPI or IGD or impulsiveness. RPI, Risk appetite index; IGD, internet gaming disorder. *P < 0.05, **P < 0.01

Analysis of variance and post hoc analysis were conducted on IGD, impulsivity, and RPI in terms of game time. The results of analysis of variance showed that there were significant differences in IGD and impulsivity between the group with game time greater than or equal to 4 h and less than 4 h and the group without playing games (*P* < 0.01). In post hoc analysis, there was a significant difference in IGD between the game group and the non game group (*P* < 0.001), and the IGD of the game group was higher than that of the non game group. In terms of impulsivity, there was a significant difference between the group with game time greater than or equal to 4 h and the group without playing games (*P* < 0.01). There was also a significant difference between the group with game time less than or equal to 4 h and the group with game time less than 4 h (*P* < 0.01). The group with game time greater than or equal to 4 h had the highest impulsivity, while the group with game time less than 4 h had the lowest impulsivity. RPI increases with the increase of game time, but there is no statistically significant difference. (Table 3; Fig. 2).

### Correlation analysis of IGD and impulsiveness and RPI

The Spearman correlation analysis was used to analyze the correlations between IGD and its six different dimensions, BIS, RPI. The results showed that there was a significant correlation between IGD and BIS, RPI (Table 4), the correlation coefficients between IGD, BIS, and RPI are 0.264 and 0.225, respectively, indicating a significant correlation. The IGD’s six different dimensions, among which all except for Mood modification are positively correlated with impulsiveness and RPI.

**Table 4:** Correlation analysis of IGD and BIS and RPI

	IGD	Salience	Mood modification	Tolerance	Withdrawal symptoms	Conflict	Relapse	
BIS	0.264**	0.245**	0.052	0.238**	0.242**	0.268**	0.244**	
Motor Impulsiveness	0.256**	0.249**	0.062	0.201**	0.249**	0.239**	0.255**	
Non-planning Impulsiveness	0.193**	0.188**	0.028	0.191**	0.166**	0.205**	0.163**	
Cognitive Impulsiveness	0.155**	0.119**	0.026	0.126**	0.134**	0.175**	0.135**	
RPI	0.225**	0.182**	0.030	0.176**	0.243**	0.283**	0.173**	

Note. IGD, internet gaming disorder; BIS, Barratt impulsiveness scale; RPI, risk appetite index.

### Mediating effects of RPI and BIS between IGD in college students

**Fig. 3:**
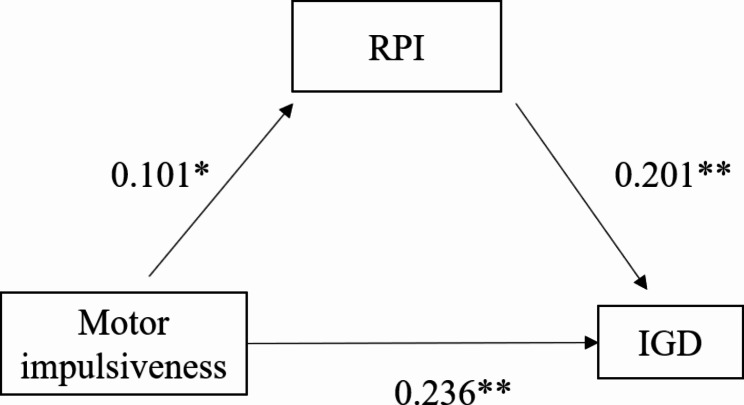
Mediation model of RPI between motor impulsiveness and IGD. Construct a mediation model with motor impulsiveness as the independent variable, IGD as the dependent variable, and risk appetite index as the mediating variable. RPI, Risk appetite index; IGD, internet gaming disorder

Firstly, IGD, RPI and motor impulsiveness are correlated with each other, indicating the possibility of further exploring the mediating effect of RPI between motor impulsiveness and IGD. Using motor impulsiveness as the independent variable, IGD as the dependent variable, and RPI as the mediating variable, a mediation model (Fig. 3) is constructed and a mediation effect analysis is conducted, with the results shown in Table 5. The Bootstrap analysis method is used to test the mediating effect of RPI between motor impulsiveness and IGD, with a confidence interval of 95% and 5000 Bootstrap samples. The results are shown in Table 6. The 95% confidence interval of the indirect effect does not include 0, indicating a significant mediating effect. The direct effect is 0.1900, the indirect effect is 0.0163, and the total effect is 0.2063, with the indirect effect accounting for 7.9% of the total effect. This indicates that RPI plays a partial mediating role between motor impulsiveness and IGD.

**Table 5:** Mediating effects of RPI and BIS between IGD in college students

Regression equation			Integration fit index				Significance of regression	
Result Variables	Prediction variables		*R*	*R* ^*2*^	*F*		*β*	*t*
IGD	Motor impulsiveness		0.26	0.07	43.63**		0.256	6.605**
RPI	Motor impulsiveness		0.1	0.010	6. 378*		0.101	2.525**
IGD	Motor impulsiveness		0.33	0.11	36.610**		0.236	6.181**
	RPI						0.201	5.264**

**Table 6:** Decomposition of total effect, direct effect and mediating effect

	Effect Value	Boot Standard Error	BootLLCI	BootULCI
Total effect	0.190	0.031	0.130	0.250
Direct effect	0.016	0.008	0.003	0.035
Total indirect effect	0.206	0.031	0.145	0.268

## Discussion

Our research focuses on the relationship between online gaming disorders, risk preference, and impulsive personality through the use of questionnaires. This study found that RPI may serve as a mediator between impulsivity and IGD, with all six different dimensions of IGD positively correlated with impulsivity and risk preference. This study provides evidence for further understanding the relationship between online gaming disorders, impulsivity, and risk preference.

We found that men scored higher on the IGD-20 scale, suggesting that men are more susceptible to developing online gaming disorder and exhibit greater gender variation. This finding substantiates the idea that men are more likely to experience IGD than women [33]. In this study, we found that men scoring significantly higher on the RPI than women. Additionally, previous research has shown that the IGD and control groups tend to make riskier decisions in the loss domain, consistent with a prior study on adaptive decision-making tasks [25, 34]. However, women tend to score higher on the impulsivity personality scale compared to men, including in areas such as non-planning impulsiveness, motor impulsiveness, and cognitive impulsiveness, and impulsive personality. It may be due to differences in personality characteristics between men and women, but woman has a strong ability to accept new things and emotional regulation and socila support. In addition, men may have a stronger spirit of risk taking, in internet games violence and aggression are more attractive to man. We established that RPI and impulsiveness play significant roles in the development of IGD.

At the same time, we analyzed the relationship between game time and IGD, impulsivity, and RPI, One-Way ANOVA revealed that IGD, impulsiveness, and control group of the gaming group with a game time of 4 h or more were statistically significant, indicating that when comparing individuals based on their gaming habits, those who spent more time playing online games exhibited higher levels of IGD and impulsiveness than those who spent less time or did not play at all. Prolonged gaming may increase the risk of gaming addiction. When a person spends a lot of time playing games, they may gradually develop a dependence on the game and cannot extricate themselves. Excessive addiction to the game can lead to difficulty concentrating, unstable emotions, and easy to make impulsive decisions, which may increase the occurrence of impulsive behavior.

Correlation analysis shows that IGD-20 and its six different dimensions, except for mood modification, are positively correlated with impulsivity and RPI. Indicating that individuals with IGD may be more prone to impulsive behavior. They may be unable to control their desires and behaviors towards games, often impulsively playing games, trying to squeeze out more time to surf the internet, while neglecting other important things. Although they consciously want to reduce their time spent on game, they were unable to succeed. Individuals with IGD are often more inclined to take risks and take risks. They may seek excitement and pleasure in the game, enjoy challenging difficult tasks, and rarely care about loss.

In the regression analysis, IGD, RPI and exercise impulse are the three variables that are correlated in pairs. Motor impulsiveness usually refers to an individual’s impulsive behavior in terms of actions. This includes an individual’s tendency to be quick, reckless, and impulsive when making decisions or taking actions. People with high scores of motor impulsivity are more likely to make quick decisions or take action when faced with risky or stimulating situations, without sufficient thinking and evaluation. In the analysis of mediating effect, RPI plays a partial mediating role in motor impulsivity and IGD. Individuals with higher RPI may be more inclined to participate in stimulating and high-risk activities, as these activities can meet their needs for adventure and stimulation. In contrast, individuals with lower RPI may be more inclined to choose safety and stability.

The Social Compensation Hypothesis suggests that individuals with higher levels of social anxiety or lower levels of social support can experience greater levels of happiness through internet usage compared to those with similar levels of social anxiety who do not use the internet [35]. This hypothesis suggests that for those facing social challenges in real life, the internet can serve as a way to escape difficulties. They may find it easier to communicate with others, establish social networks, and experience a certain level of satisfaction and happiness in the virtual environment. According to this hypothesis, individuals may face disadvantages such as poverty, loneliness, and social exclusion in their real lives, but they can gain social recognition and support in the virtual world. Individuals with high RPI are more inclined to pursue behaviors or rewards that involve higher risks and uncertainties when faced with choices. Individuals with IGD often exhibit higher risk preferences and are more likely to impulsively engage in gaming activities in virtual worlds. RPI may serve as a mediating factor between impulsivity and IGD, influencing individuals’ pursuit of rewards and supporting the social compensation hypothesis. Risk preferences may lead individuals to seek excitement and adventure in virtual worlds, engaging in social interactions to obtain more rewards and recognition. This social compensation mechanism can balance individuals’ negative emotions and social needs under unfavorable conditions in the real world, thus driving individuals’ addiction to and continued participation in the virtual world.

Impulsivity is a common feature of substance abuse, gambling, and game disorders and plays a crucial role in occurrence and maintenance of behavioral patterns. Brand et al. [36] proposed the Person-Affect-Cognition-Execution (I-PACE) model as a combinatorial process to understand the emergence and perpetuation of internet addiction disorder such as online gaming, gambling, pornography, shopping, and communication disorders. This model posits that the development of internet addiction disorder is a result of the interplay between vulnerable factors such as neurobiology and psychological characteristics, regulatory factors like coping mechanisms and cognitive biases towards the network, and intermediary factors such as context-triggered cognitive and affective responses, as well as reduced executive function. Regulatory factors such as coping mechanisms and cognitive biases towards the network can mediate the relationship between predisposing factors and internet addiction disorder. Additionally, situational stimuli such as cued responses or craving and attention bias can lead to emotional and cognitive responses that reduce inhibitory control and executive functions, promoting internet use decisions. Furthermore, conditioning processes can reinforce these relationships during addiction. The I-PACE model has significant implications for guiding future research in this area.

This paper presents some differences in comparison to the findings of previous research. However, there is common ground as many studies have demonstrated that impulsive personality is a predictive factor for IGD [4]. Moreover, prior research has indicated that young adults with IGD exhibit greater top-down goal-directed attention during decision-making tasks [25], consistent with our findings. In terms of differences, our research focuses on exploring the relationship between IGD, impulsive personality, and risky decision-making in contrast to other studies.

Indeed, several limitations were found in the present study. This study did not explore the relationship between the occurrence, symptoms, and negative consequences of gaming disorders and game types. Previous reports have shown that different games have different characteristics. It seems that players with fragile psychology are more likely to be involved in online games, especially some online game types [37]. By considering the relationship between game characteristics and player characteristics, other mechanisms of IGD development may be revealed.

Individuals with IGD may recognize the harmful effects of excessive Internet gaming, but due to their low risk aversion in the income field, they may make a risk decision and continue to play online games. To address IGD, interventions should incorporate decision-making models like the Kepner-Tregoe matrix [38], this model can promote individual thoughtful and multi criteria decision-making analysis of the negative consequences associated with excessive internet gaming. Overall, our study plays a guiding role in preventing and intervening in IGD and helps develop a more balanced perception. IGD is often associated with escapism, and it is only by confronting reality, making rational decisions, and building a healthy personality that one can better adapt to society [39, 40].

The limitations of this study include the use of a questionnaire survey, which may have introduced subjectivity into the results. It may be necessary to use further research such as experimental design to validate the findings of this study. In addition, the sample size of this study is small and limited to medical students. Therefore, future research should aim to replicate the findings of this study with a larger and more diverse sample population. Finally, our cross-sectional research design could not confirm causal relationships between risk preference and IGD. A prospective study was necessary to understand the effect of risk preference on the prognosis of subjects with IGD.

## Conclusions

Our findings suggest that there is a certain relationship between impulsivity and risk preference with IGD and its dimensions, risk preferences may function as a mediator between impulsivity and IGD. And men exhibit higher scores in IGD. This study highlights the importance of monitoring the impulsivity and risk preference of individuals who engage in internet gaming, as this may help prevent excessive gaming behavior from developing into an addiction. These findings may contribute to further research and development of intervention and prevention measures for IGD.

### Electronic supplementary material

Below is the link to the electronic supplementary material.

Supplementary Material 1: Data on college students' internet gaming disorder, impulsiveness, and risk appetite index

Supplementary Material 2: Data on college students' internet gaming disorder, impulsiveness, and risk appetite index

## Data Availability

All data generated or analysed during this study are included in this published article.
