# Supplementary material for: Association of internet gaming disorder with impulsivity: role of risk preferences
Source: BMC Psychiatry. 2023 Oct 16;23:754. doi: 10.1186/s12888-023-05265-y (PMC10580621; doi:10.1186/s12888-023-05265-y)
Supplement: Supplementary file 2 — Supplementary Material 2: Data on college students' internet gaming disorder, impulsiveness, and risk appetite index [file 12888_2023_5265_MOESM2_ESM.docx]

**Data Table Legend**

IGD, internet gaming disorder; BIS, Barratt impulsiveness scale; RPI, risk appetite index.

The IGD test includes 20 items labeled W1-W20. The BIS includes 30 items labeled B1-B30. The RPI questionnaire includes 14 items labeled H1-H14.
